# The use of mazes over time in Spanish heritage speakers in the US

**DOI:** 10.3389/fpsyg.2023.1125131

**Published:** 2023-05-31

**Authors:** Lourdes Martinez-Nieto, Theresa Moen, Melissa Pierce, Maria Adelaida Restrepo

**Affiliations:** ^1^Department of Audiology and Speech-Language Pathology, A.T. Still University, Mesa, AZ, United States; ^2^Speech and Hearing Science, Arizona State University, Tempe, AZ, United States; ^3^Department of Speech-Language Pathology, Midwestern University, Glendale, AZ, United States; ^4^Department of Communication Sciences and Disorders, University of South Florida, Tampa, FL, United States

**Keywords:** heritage speakers, Spanish in the U.S., bilingual, developmental language disorder (DLD), longitudinal, mazes

## Abstract

**Introduction:**

Mazes are linguistic disfluencies such as filled pauses, repetitions, or revisions of grammatical, phonological, or lexical aspects of words that do not contribute to the meaning of a sentence. Bilingual children are believed to increase the numbers of mazes in their native or heritage language, the minority language, as they become more proficient in the second language, the societal language. Mazes may increase over time in bilingual Spanish-speaking children as they become more proficient in English, the societal language in the United States. However, current studies have not been conducted longitudinally. Higher rates of mazes in the heritage language over time may be due to changes in language proficiency and differences in processing demands in the children as they use more complex language. Moreover, children with developmental language disorder (DLD) can also present higher rates of mazes than children with typical language. Heritage speakers, therefore, are at risk of being misdiagnosed with DLD due to high rates of mazes. Currently, we do not understand what the typical rates of mazes are as heritage speakers get older and become more proficient in the societal language. The current study examined the type and frequency of Spanish mazes longitudinally in a group of 22 Spanish heritage speakers with and without DLD and determined the changes over time.

**Methods:**

A total of 11 children with typical language development (TLD) and 11 with DLD participated in this 5-year longitudinal study. Using a wordless picture book, children completed a retelling task in Spanish during the spring of each academic year (PK to 3rd grade) as part of a 5-h testing battery. Narratives were transcribed and coded for types of mazes (filled pauses, repetitions, grammatical revisions, phonological revisions, and lexical revisions).

**Results and conclusion:**

The results of the study indicate that TLD children increased their overall percentage of mazed words and utterances. The opposite pattern was observed in the DLD group, which decreased their percentage of mazed words and utterances. In contrast, both groups demonstrated a decrease in repetitions in first grade and an increase in third grade. Additionally, the TLD and DLD children decreased in the percentage of fillers in first grade and then increased in the third grade. Results suggest that maze use is quite variable in heritage speakers and does not necessarily differentiate groups. Clinicians should not rely solely on mazes to determine ability status. In fact, high use of mazes can reflect typical language development.

## Introduction

Heritage speakers (HS) are bilinguals who are native speakers of a minority language (the home/heritage language) that was naturalistically acquired at home and who also speak the societally dominant language where they live (Montrul, 2016; Kupisch and Rothman, 2018). In our study, we focus on children who speak Spanish as the minority and heritage language within an English-speaking societal context. As of 2019, approximately 12 million children were considered HS in the US, with that number expected to grow. Of these, almost 75% speak Spanish as their home language (U.S. Census Bureau, 2016). Many HS children start their formal education as primarily Spanish speakers but rapidly switch to English dominance and Spanish becomes the Heritage Language.

Research on HS adults has reported that their grammar and fluency in the heritage language (HL) differ from those of monolinguals (Valdés, 2005; Montrul, 2016) and may resemble that of second language speakers (Bruhn de Garavito and White, 2002; O’Grady et al., 2011). Additionally, the linguistic characteristics of HS children in the HL may overlap with the linguistic profile of monolingual children of the same language with Developmental Language Disorder (DLD, formerly called specific language impairment or primary language impairment), resulting in HS children with a misdiagnosis of DLD. Understanding the development of maze use and characteristics over the course of HS’ language development and second language acquisition is critical for improving our knowledge and practices in evaluating HS with suspected DLD. In the current study, we examine the changes in Spanish maze use over time, given the limited research documenting how these characteristics change and impact children’s HL use. As children become more proficient in English, Spanish assessment is still critical as part of the whole child’s repertoire and informs accurate diagnosis.

Monolingual children with DLD exhibit significant morphosyntactic differences from children with typical language development (TLD) (Leonard, 2014). However, these differences are less clear in the case of bilingual children. Bilinguals’ linguistic characteristics often differ in fluency and morphology from monolingual speakers in the HL, which may be due to language attrition, protracted development or different developmental patterns (Morgan et al., 2013; Martinez-Nieto and Restrepo, 2021). At the same time, HS may show influences of typical second language development in English, the societal language (Paradis, 2005), as English development may be influenced by the children’s first language, Spanish. To identify the expected language characteristics of bilingual children with DLD, researchers have compared grammatical skills (Morgan et al., 2013), narrative skills (Tsimpli et al., 2016), and code-switching patterns (Gutiérrez-Clellen et al., 2009) between bilingual children with DLD and TLD. Oral language fluency, however, has received less attention in the literature, and the limited research available does not indicate clear and conclusive patterns in the use of mazes as children acquire a second language over time. Oral language fluency, in this text, refers to the linguistic flow in the children’s productions and encompasses typical disfluencies such as repetitions, revisions, and filled pauses. In addition, as children develop more proficiency in their second language and have fewer opportunities in the native language, they may present with high rates of maze use.

Some researchers have reported that an increased rate of mazes in monolingual children should be considered an indicator of DLD (Leadholm and Miller, 1995; Thordardottir and Ellis Weismer, 2002; Guo et al., 2008). However, increases in language complexity correlate with an increase in mazes (MacLachlan and Chapman, 1988; Rispoli and Hadley, 2001; Carias and Ingram, 2006) and are therefore expected as children’s language naturally develops and becomes more complex. These ambiguities and the limited extant research make typical or linguistic-based disfluencies, specifically mazes, an important area of research for helping to differentiate DLD from TLD in young HS.

### Mazes

Mazes are linguistic non-fluencies, such as fragments of word(s) that are not part of the intended message (Loban, 1976; Levelt, 1989). Studies have varied on the terms used to refer to mazes (revisions, interruptions, speech disfluencies, circumlocutions, hesitations, communication breakdowns, and self-corrections). In the present study, we will refer to them as mazes. Mazes are typically grouped into types such as filled pauses, repetitions, and revisions (phonological, lexical and grammatical–DeJoy and Gregory, 1985; Dollaghan and Campbell, 1992; Bedore et al., 2006). These maze types typically fall into two overarching categories: fillers (filled pauses and repetitions) or content (grammatical, lexical, and phonological revisions) mazes. According to Thordardottir and Ellis Weismer (2002), speakers use repetitions or filled pauses as a pragmatic function and do not change the intended meaning of the utterance, while revisions (phonological, grammatical, or lexical) may be part of processing demands and alter the meaning of the sentence. Rispoli (2003) and Rispoli et al. (2008) propose an explanation for the difference between fillers (stalls) and content (revisions) mazes. They state that stalls/fillers are due to glitches that are temporary problems while encoding the message. On the other hand, they attribute revisions to a self-monitoring process. While stalls allow the speaker to wait for the following encoding processes, revisions work as a way to compare the intended message to the actual linguistic output. This is an important distinction because revisions may be considered indicators of grammatical knowledge.

Mazes are present in typical language development in all languages, and all speakers produce mazes from childhood through adulthood. Research has reported that maze frequency correlates with linguistic and grammatical complexity. For example, higher rates of mazes are observed when sentence length increases (MacLachlan and Chapman, 1988; Rispoli and Hadley, 2001; Carias and Ingram, 2006) and when grammatical skills increase (Rispoli, 2003). Mazes are more common in narration, a more complex task, than in conversation (Leadholm and Miller, 1995; Bedore et al., 2006; Wetherell et al., 2007). For example, Rispoli and Hadley (2001) investigated how sentence complexity may determine maze production. They examined maze production in a group of 26 TLD children (ages 2; 6 to 4). The results showed that children had more mazes in longer and more complex sentences. More recently, Rispoli (2018) reported that when 27-month-old children used a more diverse set of sentence subjects during play interaction with their mothers, they also used more revisions.

Maze rates are also correlated with age and language proficiency. For example, in typically developing children, mazes are expected to decrease with age as a sign of language maturity and better proficiency (MacLachlan and Chapman, 1988; Rispoli and Hadley, 2001; Carias and Ingram, 2006). Consistent with this expectation, Loban (1976) reported lower rates of mazes in a group of 35 English-speaking children who were considered effective or proficient language users. However, if children show formulation problems and do not attempt to reformulate or repair them, it may indicate processing difficulties (Kaur et al., 2011).

### Maze production in heritage speakers

Heritage speakers’ maze rates and types may be different across their two languages (Bedore et al., 2006; Byrd, 2018). The nature of bilingual acquisition imposes different processing demands for each language in bilingual children depending on their proficiency in the language they are using, which is not static and changes dynamically throughout their development. These differences in processing demands may manifest in the use of mazes.

In adult bilingual speakers, mazes are more frequent in the second language than in the native language when the native language is dominant (Rieger, 2003). Studies show that all bilingual and monolingual children produce mazes within narrative contexts, providing valuable information within a naturalistic, functional task with a processing demand over and above that of conversation (Fiestas et al., 2005; Bedore et al., 2006; Taliancich-Klinger and Bedore, 2019).

Research with typically developing Spanish-English bilingual children in the early school years found no significant differences in overall maze production within narrative contexts between monolingual and bilingual children in English or Spanish, suggesting that their maze use was not related to bilingualism (Fiestas et al., 2005; Bedore et al., 2006; Taliancich-Klinger and Bedore, 2019). As mentioned, these studies have focused on comparing young HSs’ maze production with that of their monolingual peers in English and Spanish. Therefore, there is a need to examine longitudinal changes in young HS’ maze production in the HL, which will contribute to our understanding of children’s maze use patterns over time and how typical HS language differs from those with DLD.

### Mazes and developmental language disorder

Research with monolingual Spanish-speaking children with DLD shows that they have greater rates of mazes than TLD children and that the types of mazes they use include more diverse linguistic elements. Jackson-Maldonado et al. (2013) conducted a study with 10 children (5 TLD and 5 DLD) monolingual Spanish-speaking children (5–9 years of age). They found that in a narrative retelling task, TLD children used mainly lexical mazes with nouns [e.g., (la ra*) Irvin aventó a la rana “(the fro*) Irvin threw the frog”], while DLD children used repetitions and grammatical mazes related to clitics, prepositions, and determiners [e.g., *para va a picarle (una)la Ø(s)abejas “to go to sting him (a) the bees”]*, all of which are vulnerable and prone to errors for this group. Research on mazes in HS is limited, and development patterns through the years will not necessarily reflect those described above. Research in this area is still very limited cross-linguistically and with bilingual populations.

In English-speaking children, researchers have found similar results as reported above, within sentence contexts (Boscolo et al., 2002; Finneran et al., 2009), narrative contexts (MacLachlan and Chapman, 1988; Thordardottir and Ellis Weismer, 2002; Guo et al., 2008), and conversational contexts (MacLachlan and Chapman, 1988). However, findings are equivocal regarding the maze types used among the different ability groups. For example, MacLachlan and Chapman (1988) found no difference in the types of mazes produced by 9–11 year-old TLD children and those with DLD, while Finneran et al. (2009) found that 8-year-old children with DLD produced significantly more repetitions than their TLD peers. Moreover, Boscolo et al. (2002) found that, on average, 9-year-old children with previous diagnoses of DLD produced more whole-word and phrase repetitions, revisions, and filled pauses and significantly more part-word repetitions than their TLD peers. Hodge et al. (1999) found that toddlers with DLD produced significantly more part-word and whole-word repetitions than their TLD peers. In contrast, two studies have shown that children with DLD actually produce the same or fewer mazes compared to their TLD peers. Merits-Patterson and Reed (1981) found no difference in quantity or type of mazes between TLD and DLD preschoolers not receiving treatment. Meanwhile, Thordardottir and Ellis Weismer (2002) found that children with DLD actually used fewer filled pauses than the TLD children.

There are few studies on maze production in general, and even fewer involving HS school-aged children (Fiestas et al., 2005; Bedore et al., 2006; Carias and Ingram, 2006; Kaur et al., 2011; Taliancich-Klinger et al., 2013, 2021; Byrd et al., 2015; Taliancich-Klinger and Bedore, 2019; Rojas and Irani, 2020). No studies to date, as far as we know, have examined maze production longitudinally in young HS with TLD and DLD. Therefore, the present study will help us better understand what typical maze use development looks like in young HSs as they increase their second language proficiency over time. Specifically, we test the hypothesis that as children increase their second language proficiency, the percentage of mazes in the HL increases. The present study aims to contribute to the knowledge base regarding Spanish HS development and the use of mazes in the United States’ English-speaking societal context. In addition, this study will contribute to our understanding of how to differentiate TLD from DLD in Spanish as a heritage language.

## The current study

This study aimed to examine the overall use of mazes and the type and frequency of mazes longitudinally in a group of Spanish-HS with and without DLD during preschool, first, and third grade. Due to the assumption that mazes in Spanish, the heritage language (HL), increase as English proficiency improves, we focused on the following research questions:

(1) What is the overall amount of maze production in Spanish per language ability group (HS-DLD/HS-TLD) and grade (Pre-K, 1st, 3rd), and are there differences between groups and grade?

- We hypothesize that the number of mazes produced will be higher overall for children with DLD. Further, we hypothesize that the mazes will decrease over time for the HS-TLD group, but be stable or increase by grade for the HS-DLD group.

(2) What specific types of mazes (i.e., revisions, repetitions, etc.) do Spanish-HS produce in Spanish over time?

- We hypothesize that filler mazes, such as filled pauses and repetitions, will be more common than content mazes, such as revisions, for both groups.

(3) As children increase proficiency in English, are there differences in the frequency and types of mazes used in Spanish by grade and by ability group (HS-DLD/HS-TLD)?

- We hypothesize that as children increase English proficiency, their maze use in Spanish will increase or remain consistent for HS-DLD children and that it will reduce over time for HS-TLD children. We also predict that differences will arise in content vs. filler mazes, with content used more frequently by the TLD group and filler used more frequently by the DLD group.

## Materials and methods

### Participants

Participants in this study are part of the Language and Reading Research Consortium (LARRC) study, which had two samples of children, one Spanish-English bilingual and one English monolingual. The current study addresses the language of twenty-two participants from the 285 bilingual children that started in PreK and spoke Spanish at home. The sample consisted of 11 Spanish-speaking children with TLD and 11 Spanish-speaking children with DLD who were recruited in preschool and followed through third grade. Measures for qualification in this study included the *Clinical Evaluation of Language Fundamentals*- Preschool, 2nd Edition–Spanish (CELF-P2 Spanish), a norm-referenced standardized measure with bilingual children in the US (Wiig et al., 2009). This measure includes four core subtests that assess grammar, morphology, and semantics. In addition, we used the Spanish Screener for Language Impairment in Children (SSLIC, Restrepo et al., 2010). This measure provides a subtest in morphology, sentence repetition and non-word repetition, developed and standardized with over 650 bilingual children in the greater Phoenix area. All participants met the following inclusionary criteria: (a) parents reported that their child spoke Spanish as their native language at home at least 50% of the time; (b) parents and teachers reported the child spoke more Spanish than English; (c) child had no severe speech, language, cognitive, sensory, or motor disabilities that would preclude participation in assessments per parent and teacher report; (d) child was attending preschool, and was eligible to enter kindergarten the following year.

Children were screened in Spanish in PreK when they spoke mostly Spanish. In subsequent years they were evaluated in both languages. Children with DLD were identified in Pre-Kindergarten using (a) the CELF-P2 Spanish by scoring below 7 on the word structure and recalling sentences subtests; (b) scoring below 11 (out of a possible 44) on the SSLIC measure; (c) parents reported language concerns; and (d) whether they were receiving language services. All children were followed for 5 years. Children’s schooling in kindergarten through 3rd grade was in English only due to Arizona state laws at the time of the study (AZ Proposition 203, passed in 2000). Bilingual children in our study were in such English-only classrooms through elementary school. Therefore, exposure to Spanish only occurred outside the school.

For inclusion in our analyses, we randomly selected the TLD children from those in the database with the most complete data for the 3 years under study (Pre-K, 1st, and 3rd grades). Inclusion criteria for HS children with DLD required complete data. In cases of code-switching, we required transcripts to have at least 10 sentences in Spanish to be considered complete data for each time point. Participants’ demographics are shown in Table 1.

**TABLE 1:** Participants demographic Information.

	TLD	DLD
Total	11	11
Female	5	4
Male	6	7
Age in months at PK (Mean)	59	59
Word structure^+^** (Mean)	9	4.1
Recalling sentences^+^** (Mean)	9.5	5.8
SSLIC*/** (Mean)	25	5
Language services	None	All

^+^Scaled scores from the clinical evaluation of language fundamentals-2 (Spanish) **p < 0.001.

*Raw score from SSLIC-screener for Spanish speaking children.

### Materials

As part of the larger study, children participated in a 5-h battery that included oral language and literacy measures. The sessions consisted of 45 min to an hour and a half, depending on the grade and the child’s attention, with breaks during this time if needed. The language samples included one of the Mercer Mayer frog wordless picture books: *Frog on his own* and *A boy a dog a frog and a friend* (Mayer, 1967, 1973). The Spanish samples came from retelling one of the two Spanish frog stories with a Spanish tester. We used a story script that we created for each story, controlling for length and lexical diversity to be equivalent across the wordless books used in the longitudinal study. The examiner read the story out loud to the child, and then the child retold the story as they went page by page. Retelling was used because we started the protocol in preschool when many children may not have experience telling stories, and we wanted to maintain a consistent protocol year to year. The stories alternated between the two books from year to year. So, every other year, the child retold the same story. The stories also changed by language. A native speaker of the language assessed children in only one language per day. If a child was seen twice in 1 day for some exceptional reason, different assessors evaluated the child in the different languages.

### Procedures

Using a wordless picture book, children completed a Spanish oral narrative retelling task during the spring of each academic year (PK to 3rd grade) as part of a 5-h testing battery for the larger study. Narratives were transcribed and coded for types of mazes. Specifically, the mazes were coded as follows: Filled pauses, which are non-linguistic vocalizations [e.g., *el niño (uhm) vio a la (uhm) rana* “the boy (uhm) saw the (uhm) frog”]; repetitions, which are part-word, whole word, or phrases that the speaker repeats with no additional meaning [e.g., (*el*) *el perro se fue* “(the) the dog went away”]. Revisions were categorized into lexical, phonological and grammatical. Lexical revisions involve changes of the word choice (e.g., *el sapo/la rana se fue–*the toad/the frog left), lexical revision with code-switching involved changes of the word choice with a change in language [e.g., (*el* dog) *el perro se fue*–“the dog went away”], phonological revisions are the correction of sounds of the word [e.g., *el perro se fue (tras) atras “*the dog went (bek) behind”] and grammatical revision involved changes in the grammatical structure of the sentence, such as gender agreement, subject-verb agreement, or word order (*el rana/la rana se fue* “the frog left”–masculine to feminine article). We analyzed the samples using the Systematic Analysis of Language Transcripts software- research version 20 (SALT; Miller and Iglesias, 2020). The percentage of specific maze types was calculated based on the total number of mazes produced by the child in the whole sample. If the child produced a total of 20 mazes in the narrative and 10 were repetitions, repetitions represented 50% of the total number of mazes. The same procedure was used for each type of maze. The percentage of mazed utterances per sample was calculated by including any utterance with at least one maze. The denominator was the total number of utterances in the narrative. This was used rather than the total number of mazed utterances because samples varied in length, allowing us to consider the more comparable proportion of mazes in each sample. In addition to the summary codes described above, we obtained measures of mean length of utterance, number of different words, percent of ungrammatical sentences, total number of sentences and number of mazed sentences.

### Analyses

Typically, group differences over time are evaluated using analysis of variance (ANOVA). For this study, a Linear Mixed Model (LMM) was chosen for several reasons. First, this method of analysis accounts better for the small sample size by including individual participants as random effects, thus retaining more statistical power. LMM also deals better with non-independent samples by explicitly accommodating dependency between observations from the same participant (Breslow and Clayton, 1993; Krueger and Tian, 2004; Aarts et al., 2014). LMM maximizes power by using the data in the long form and handles missing data using maximum likelihood estimation rather than list-wise deletion, thus retaining more student outcomes at each time point (Krueger and Tian, 2004). Additionally, it allows us to consider fixed factors, which are sampled from the population, and random effects, which are associated with individual experimental units randomly drawn from the population (Gelman and Hill, 2007; Magezi, 2015). SPSS Version 28 was used for all analyses. Results are reported as *F* statistics, significance (set at *p* < 0.05), and partial eta squared effect sizes (Cohen et al., 2002). The fixed factors were the grade (Preschool, 1st or 3rd) and group (TLD vs. DLD). Individual students were treated as random factors. A Bonferroni correction was applied to each LMM to adjust for multiple comparisons.

## Results

To answer the first research question of the changes in the use of overall mazes over time by group, we examined differences in the overall percentage and types of mazes used by bilingual Spanish HS children with and without DLD who were attending English-only schooling. Descriptive statistics for oral language production measures, such as MLU, are found in Table 2. These measures are reported to give context to the specific maze production results and show overall language development trajectories. Additionally, descriptives on the maze types are included in Table 3 as the percent of total mazes.

**TABLE 2:** Oral language production descriptive statistics [Mean (SD)].

	PK		1st grade		3rd grade	
	**HS-TLD**	**HS-DLD**	**HS-TLD**	**HS-DLD**	**HS-TLD**	**HS-DLD**
TNW	128.8 (52.6)	98.3 (49.8)	233.8 (74.5)	197.8 (90.5)	325.5 (89.9)	268.5 (127.5)
MLU	5.2 (1.1)	4.4 (1.3)	7.3 (0.9)	6.6 (1.1)	8.1 (0.8)	7.4 (1.0)
NDW	55.4 (18.4)	40.1 (13.2)	77.0 (17.4)	70.6 (17.7)	92.3 (18.3)	73.3 (31.2)
Maze words (%)	13.9 (4.8)	23.3 (9.5)	16.4 (7.0)	17.2 (8.3)	16.0 (7.1)	13.4 (6.7)
Total utterances	24.2 (9.6)	21.1 (9.4)	31.6 (10.0)	31.0 (13.8)	41.1 (12.3)	36.0 (17.0)
Mazed utterances (%)	41.0 (12.0)	56.0 (17.0)	52.0 (18.0)	48.0 (16.0)	58.0 (23.0)	44.0 (26.0)

TNW, total number of words; MLU, mean length of utterance -words; NDW, number of different words; CS, code-switching.

**TABLE 3:** Percentage of maze production by type descriptive statistics [Mean (SD)].

	PK		1st grade		3rd grade	
	**HS-TLD**	**HS-DLD**	**HS-TLD**	**HS-DLD**	**HS-TLD**	**HS-DLD**
Filled pauses	23.9 (21.0)	16.7 (15.4)	19.6 (22.9)	20.7 (21.8)	16.0 (16.8)	19.9 (15.8)
Repetitions	53.5 (19.5)	63.1 (20.6)	43.7 (20.9)	43.0 (11.8)	51.0 (12.6)	57.2 (19.2)
Grammatical revisions	11.3 (9.5)	11.2 (11.6)	16.6 (12.2)	12.4 (14.3)	14.5 (8.8)	9.7 (11.2)
Phonological revisions	0.4 (1.4)	0.0	0.3 (0.9)	0.8 (2.0)	0.0	0.0
Lexical revisions	10.9 (10.1)	7.9 (9.8)	19.5 (10.1)	21.1 (13.1)	18.4 (14.0)	13.3 (4.2)
Lexical revisions w/CS	0.0	1.1 (3.2)	0.3 (0.9)	2.0 (2.7)	0.1 (0.4)	0.0
Content mazes	22.6 (10.5)	20.2 (11.1)	36.7 (15.2)	36.3 (17.8)	33.1 (16.8)	22.9 (6.8)
Filler mazes	77.4 (10.5)	79.8 (11.1)	63.3 (15.2)	63.7 (17.8)	66.9 (16.8)	77.1 (6.8)

Percentages are based on the total number of mazes. Content mazes = all revision types; Filler mazes = repetitions and filled pauses.

To answer research question one: “What is the percent of mazed utterances used over time by children with TLD and DLD?” LMM results showed a significant main effect of grade with a medium effect size [*F*(2,274.61) = 11.42, *p* < 0.001, η^2^ = 0.07], but not group membership [*F*(1,17.08) = 1.02, *p* = 0.33, η^2^ = 0.06], though group membership did show a medium effect size (≥0.06; Cohen et al., 2002). A significant grade-by-group interaction effect was also observed, with a large effect size [*F*(2,274.61) = 33.94, *p* < 0.001, η^2^ = 0.20] (≥0.14; Cohen et al., 2002). Further probes of mean differences revealed that overall, maze use significantly increased for HS-TLD children from preschool to first grade, followed by a non-significant decrease from first to third grade. Table 4 displays mean differences in the percentage of mazes used within groups across years of the study for each individual type of maze, and for the two maze categories (fillers and content). These means are derived from the averaged random effects across subjects. Overall percentage of mazed utterances decreased significantly across the grades for HS-DLD.

**TABLE 4:** Mean differences in percentage of mazes across samples by year.

	Pre-K to 1st		1st to 3rd		Pre-K to 3rd	
	**HS-TLD**	**HS-DLD**	**HS-TLD**	**HS-DLD**	**HS-TLD**	**HS-DLD**
Overall maze production SE 95% CI	0.03* 0.01 0.004 −0.05	−0.06* 0.01 −0.09 to −0.04	−0.004 0.01 −0.03 −0.02	−0.04* 0.01 −0.07 to −0.003	0.02 0.01 −0.002 −0.05	−0.10* 0.01 −0.13 to −0.07
Filled pauses SE 95% CI	−4.26 2.54 −10.39 to 1.86	1.09 3.14 −6.47 to 8.64	−7.67* 2.87 −14.58 to −0.77	10.10* 4.17 0.07–20.14	−11.94* 2.87 −18.85 to −5.03	11.19* 3.94 1.70–20.69
Repetitions SE 95% CI	−9.81* 2.11 −14.88 to −4.74	−15.92* 2.60 −22.18 to −9.65	9.91* 2.38 4.18–15.63	2.28 3.47 −6.07 to 10.62	0.10 2.38 −5.63 to 5.82	−13.64* 3.27 −21.53 to −5.75
Grammatical revisions SE 95% CI	5.35* 1.51 1.70 –8.99	0.67 1.87 −3.81 to 5.17	−2.35 1.71 −6.46 to 1.76	−1.87 2.47 −7.82 to 4.08	3.00 1.71 −1.12 to 7.12	−1.20 2.34 −6.83 to 4.44
Phonological revisions SE 95% CI	−0.13 0.14 −0.47 −0.22	0.74* 0.18 0.31–1.17	−0.40* 0.16 −0.79 to −0.01	−0.48 0.24 −1.05 −0.09	−0.53* 0.16 −0.92 to −0.14	0.26 0.22 −0.28 −0.80
Lexical revisions SE 95% CI	8.56* 1.56 4.81–12.32	12.80* 1.92 8.18–17.42	0.64 1.75 −3.58 to 4.86	- 8.37* 2.53 −14.46 to −2.29	9.21* 1.75 4.99–13.42	4.43 2.40 −1.35 to 10.20
Lexical revisions with CS SE 95% CI	0.28 0.21 −0.23 −0.80	0.83* 0.26 0.20–1.47	−0.19 0.24 −0.77 −0.39	−2.20* 0.35 −3.04 to −1.36	0.09 0.24 −0.49 to 0.67	1.37* 0.33 −2.16 to −0.57
Filler mazes SE 95% CI	14.07* 1.88 −7.29 to 2.97	15.00* 2.33 9.40–20.61	−2.16 2.13 −7.29 to 2.97	−12.89* 3.09 −20.22 to −5.45	11.91* 2.13 6.79–17.04	2.11 2.93 −4.93 to 9.16
Content mazes SE 95% CI	−14.06* 1.89 −18.61 to −9.52	−14.99* 2.33 −20.60 to −9.38	2.16 2.13 −2.97 to 7.28	12.91* 3.09 5.47–20.35	−11.91* 2.13 −17.04 to −6.78	−2.08 2.93 −9.12 to 4.97

*p < 0.05; SE, standard error; CI, confidence interval. Reference groups are the TLD group in each grade. These mean differences are derived from the averaged random effects across subjects.

**FIGURE 1:**
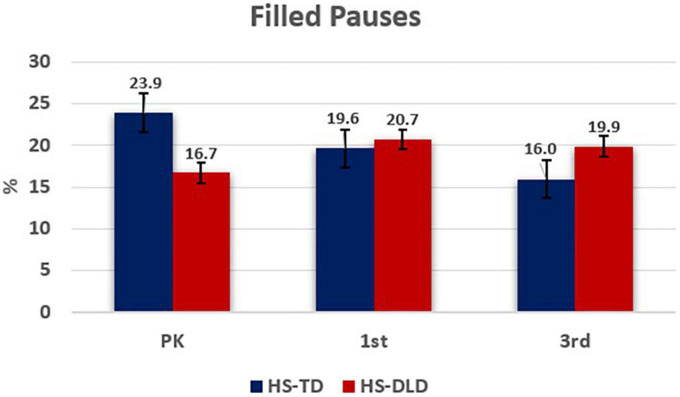
Filled pauses by grade and group.

To answer research questions two and three about the types of mazes that HS use over time and if there are differences between HS-TLD and HS-DLD in the types of mazes used, we analyzed the maze types individually using an LMM for each type. For filled pauses, grade [*F*(2,275.29) = 0.32, *p* = 0.73 η^2^, = 0.002] and group [*F*(1,16.82) = 0.13, *p* = 0.72, η^2^ = 0.01] effects were not significant and showed small effect sizes (<0.06; Cohen et al., 2002). Nonetheless, a significant grade-by-group interaction effect was observed with a medium effect size [*F*(2,275.29) = 11.35, *p* < 0.001, η^2^ = 0.08]. Based on further analysis of mean differences, this effect was driven by group differences between first and third grade, with the HS- DLD group increasing their use of filled pauses while the HS-TLD group decreased their use (Table 4). A visual representation is shown in Figure 1.

**FIGURE 2:**
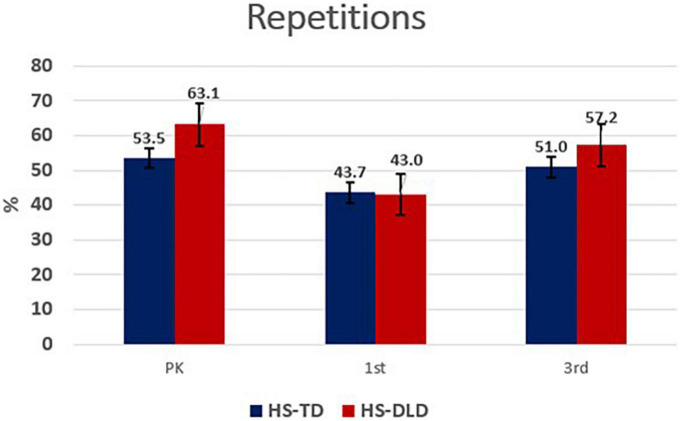
Repetitions by grade and group.

Analysis of the use of repetitions also revealed significant differences by year [*F*(2,273.90) = 29.74, *p* < 0.001, η^2^ = 0.18] and a year by group interaction [*F*(2,273.90) = 5.96, *p* < 0.05, η^2^ = 0.04], with large and small effect sizes, respectively. Upon probing mean differences, the interaction appears driven by both groups’ reduced use of repetition between preschool and first grade, followed by increased use between first and third grade (see Table 4). A visual representation is shown in Figure 2.

**FIGURE 3:**
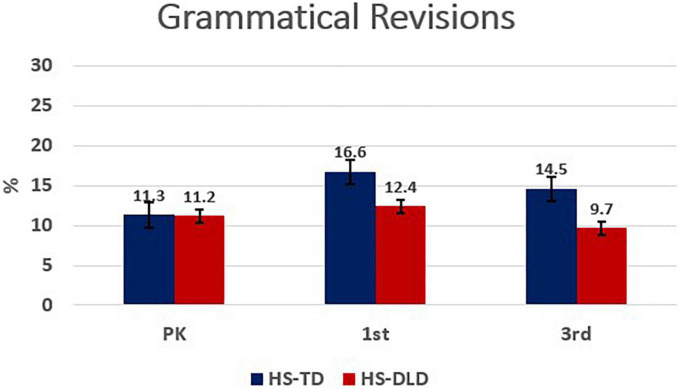
Grammatical revisions by grade and group.

Analysis of grammatical revisions showed a significant main effect for grade with a small effect size [*F*(2,277.05) = 3.19, *p* = 0.04, η^2^ = 0.02]; however, group differences were not significant, nor was there a group by grade interaction effect. A significant increase in the use of grammatical revisions by the HS-TLD group between preschool and first grade appeared to drive the main effect for grade (see Table 4). A visual representation is shown in Figure 3.

Analysis of phonological revisions showed significant differences by grade [*F*(2,276.09) = 5.82, *p* = 0.003, η^2^ = 0.04], and a group by grade interaction effect [*F*(2,276.09) = 8.47, *p* < 0.001, η^2^ = 0.06], with small and medium effect sizes, respectively. Further examination of mean differences showed that children in the HS-DLD group significantly increased their use of phonological revisions between preschool and first grade, while those in the HS-TLD group significantly decreased their use between first grade and third grade (Table 4). Despite these significant main effects, phonological revisions represented a very small proportion of overall mazes used by both groups in all grades. Due to the small proportion, a visual representation is not provided.

**FIGURE 4:**
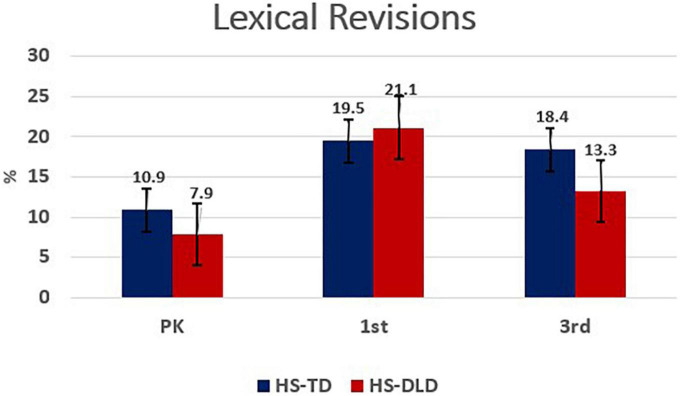
Lexical revisions by grade and group.

Analysis of lexical revisions showed a significant grade [*F*(2,278.22) = 38.46, *p* < 0.001, η^2^ = 0.22] and group-by-grade interaction [*F*(2,278.22) = 4.40, *p* = 0.01, η^2^ = 0.03] with large and small effect sizes, respectively. Group differences were not significant. Further probing of mean differences revealed significant increases between preschool and first grade for both HS-TLD and HS-DLD groups and a significant decrease between first grade and third grade for the DLD group exclusively (Table 4). A visual representation is shown in Figure 4. Lexical revisions with code-switching were also analyzed and showed main effects for grade [*F*(2,275.81) = 16.24, *p* < 0.001, η^2^ = 0.11] and a group by grade interaction [*F*(2,275.81) = 11.31, *p* < 0.001, η^2^ = 0.08], both with medium effect sizes. Probes of mean differences showed an increase from Preschool to first grade, followed by a decrease from first to third grade for the HS-DLD group exclusively. The HS-TLD group did not show significant changes between any grades. Like phonological revisions, lexical mazes with code-switching represented a minimal portion of overall mazes used, therefore, a visual representation is not provided (see Table 4).

Also, of interest for this research was the use of content mazes (grammatical, phonological, and lexical) and filler mazes (filled pauses and repetitions, Thordardottir and Ellis Weismer, 2002). Descriptive analyses showed that TLD and DLD children used filler mazes more frequently than content mazes overall, with repetitions as the most frequent type (Table 3). In both groups, content maze use increased between preschool and first grade, followed by a decrease. Filler mazes, on the other hand, decreased for both groups from preschool to first grade, followed by an increase in third grade. Interestingly, third grade students with DLD mirror maze production for TD children in preschool, showing similar values for content and filler mazes. Combined results indicate that amongst content mazes, phonological revisions were the least frequent. In the TLD group, grammatical and lexical revisions were similar in frequency in preschool while in the DLD group, grammatical revisions were more frequent than lexical revisions.

Finally, we analyzed the filler and content maze categories using LMM. For filler mazes, the analysis revealed significant increases between preschool and first grade for both DLD and TLD groups. For children with DLD, a decrease between first and third grade was also significant, For content mazes, a decrease in use between preschool and first grade was significant for children with DLD and TLD. For those with DLD, the increase between first grade and third grade was also significant, though this was not true for children with TLD (Table 4).

## Discussion

The purpose of the current study was to examine the use of mazes over time by grade (PreK, 1st, and 3rd grade) and ability group (DLD, TLD) of Spanish HS attending English-only instruction in public schools in Arizona. Also, we examined the specific types of mazes used over grade by ability groups. Our results indicate that children differed in the number of mazes used and the specific types between groups and across grades. In addition, results show that all children increased their Spanish total number of words, number of different words, and mean length of utterance over time despite being in a subtractive language environment (Paradis, 2010; Thordardottir, 2015; Paradis et al., 2021). Exposure to English-only education did not necessarily lead to language loss (Montrul, 2016) and these results support protracted but continued HL development (Martinez-Nieto and Restrepo, 2021).

### Maze performance by grade

Results by grade indicate that the use of mazes is dynamic and affected by variation in language experience. For example, TLD children increased the overall percentage of mazes between Pre-K and 1st grade, followed by a non-significant decrease between first and third grade. In contrast, DLD children decreased the overall percent of mazes used. Patterns also emerged in children’s use of specific maze types. We found variability between preschool and third grade when analyzing the specific quantity and quality of mazes used by grade. For example, repetitions and filled pauses are considered filler mazes rather than content mazes in that they do not add to or correct the meaning of the sentence when used; filler mazes were used most frequently across groups and grades. In contrast to the overall maze use, repetitions decreased from PreK to first grade, and increased from first to third for both groups. Bedore et al. (2006) suggested that the use of repetition was not related to language proficiency, as monolingual and bilingual children used comparable amounts of mazes. Results of the present study coincide with this finding. As English proficiency increased and children spent more time in English schooling, repetitions were consistently the most frequent regardless of grade or ability status.

For both groups over time, lexical revisions showed the same pattern as overall maze use in that they increased from PreK to first grade and decreased from first to third, and these did not differ between groups. Phonological and grammatical revision mazes were used the least frequently, along with code-switching lexical revision mazes. Despite infrequent use, the pattern for grammatical mazes increased initially, followed by a decrease in both groups, similarly to the use of repetitions.

### Maze performance by ability group and type

In general, ability groups differed in two significant ways in their maze use by third grade. The TLD group produced more content mazes in third grade compared to the DLD group, while the DLD group used more filler mazes despite similar usage between groups in the earlier grades. These results suggest that the use of content mazes may show more metalinguistic awareness in that TLD children are able to identify and self-correct lexical and grammatical errors while their DLD peers do not have this skill. On the other hand, the high use of filler mazes in both groups suggests an over-reliance on pragmatic strategies to maintain communication. This may reflect language production difficulties associated with accessing the right word, language ability, or both. Filler mazes may result from difficulty with processing demands, in which children are taking time to formulate the language needed to express themselves. That is, the fillers allow the child to maintain the flow of discourse as they formulate the sentence (Rispoli, 2003). This contrast is notable because HS children decrease the use of filler mazes in first grade, and increase again in third, maybe reflecting the changes in school demands as time in school may decrease the amount of time speaking Spanish. These results contrast with those of Thordardottir and Ellis Weismer (2002) who found that DLD children produced a lower number of filler mazes than TLD children. Current results suggest that the use of content mazes is a sign of typical language development in HS. Similarly, Rispoli (2003, 2018) found that monolingual speakers increased their use of content mazes as their language became more complex. Increases in language complexity correlate with an increase in mazes (MacLachlan and Chapman, 1988; Levelt, 1989; Rispoli and Hadley, 2001), and therefore are part of typical language development (Loban, 1976), whether they are monolingual or bilingual. For example, Loban found great variability in the use of mazes in TLD children, with some having high maze percentages, while others having lower percentages of mazes in their conversations and narratives. Rispoli (2003, 2018) found that when children produced more complex syntax and higher lexical diversity, they produced more revisions; however, these studies were looking at the language of very young children. Within their sample of children with ADHD, Bangert and Finestack (2020) found that higher expressive language ability was related to increased filler mazes, and higher MLU was related to increased revisions, repetitions, and content mazes.

The mean percent of mazed utterances was lower for TLD children at the start of the study (see Table 2) compared to the DLD group. By the end of study, the TLD group had the same percentage of mazed words as at the beginning of the study and the DLD group had a lower percentage than at the beginning of the study. The DLD children decreased the percent of mazed utterances over time, but as we discuss below, it is possible that this reflects less awareness of their mistakes. This concurs with Thordardottir and Ellis Weismer (2002) who found that DLD children used less filled pauses than typical children in general.

In our study, DLD children produced similar or more filled pauses than TLD children, although those with DLD slightly decreased their use of filled pauses from first to third grade. Those with TLD decreased their use from Pre-K to first grade, and from first to third grade. Thordardottir and Ellis Weismer (2002) speculated that filled pauses, a type of filler maze, serve a different function than content mazes and, therefore, may be less impacted by language-based deficits in children with DLD.

Our findings may reflect an increase in children with DLDs’ ability to compensate for language deficits by giving themselves more time to speak through the use of filled pauses, a pragmatic strategy on which they may rely more as they get older and develop social skills. The TLD group, in contrast, may not rely on this type of pragmatic strategy as much, because they are less likely to have difficulty producing the HL in the first place. Loban (1976) argued that mazes are not necessarily indicative of typical or disordered language and are present in high- and low-ability groups. Therefore, mazes may reflect highly complex and less fluent language, depending on the maze manifestation, but this dysfluent language does not differentiate typical or DLD language. On the other hand, the limited use of mazes can reflect thoughtful, well-planned language or it can reflect low-language ability reflecting limited awareness of their mistakes and lexical difficulties. Therefore, mazes alone are not a good measure to identify DLD given that mazed language also comes naturally with more complex typical language. HS Spanish skills may deteriorate with limited use in the English-only academic environment, or they may improve. Interestingly, particularly for the DLD group, reduction in mazes may indicate less awareness of errors. For example, the TLD group increased the percentage of mazed words and mazed utterances whereas the DLD children decreased in both. These distinctions do not assist speech-language pathologists in differentiating whether a HS child’s maze production in the HL suggests a DLD or whether it reflects expected patterns in typical language development. For example, if a third-grade bilingual child produces mazes frequently when telling a story in the HL, and these mazes are often fillers, rather than revisions, this could align with reduced skills in the HL due to DLD or attrition from limited use of the language. Therefore, detailed language use history information on the child’s HL input and examination of other language sample measures such as MLU, subordination index, and grammaticality would help make the distinction given the variability of mazes in the bilingual population.

In terms of type by group, the TLD group used more grammatical revision mazes overall than the DLD group, contrasting the findings of Jackson-Maldonado et al. (2013) who reported that DLD Spanish–speaking children produced more mazes in determiners and pronouns than their peers with TLD. These differences across studies may reflect the differences between monolinguals and HS. Many of these revisions involved gender agreement errors for articles such as *el, la, los* and *las*, which are often reported in HS, but not in monolingual speakers. It may be that the DLD group used fewer grammatical mazes because they were not aware of making these gender agreement errors in article use, and therefore did not self-correct with a maze. These results suggest that the TLD group improved their linguistic awareness, showing they could notice and correct these errors because of their more mature linguistic system, which is reflected in longer and more complex utterances in Spanish and a higher number of different words (Table 2). These observations concur with Restrepo and Gutiérrez-Clellen (2001) who showed that articles are vulnerable to errors in Spanish HS children with DLD. Despite this observation, the present study did not show a significant interaction effect for grade and group for grammatical revision mazes.

The children differed in the use of content mazes by ability group in third grade. Children with DLD increased the use of content mazes in first grade and significantly decreased in third grade while the TLD children slightly decreased in third grade. This same pattern was observed when we examined lexical and grammatical revisions. Lexical revision mazes were more frequently used than the other types of content mazes for both groups, and also showed a group by grade interaction effect. These mazes increased significantly for both groups between preschool and first grade but decreased significantly for the DLD group only between first and third grade. Overall, the TLD group used a larger quantity of lexical revisions. Like grammatical revision mazes, this higher usage in TLD children may reflect greater proficiency and self-monitoring than the DLD group has, such that they are able to recognize when they have made an error and correct it with a revision. On the other hand, the DLD group does not recognize the error in the first place and simply continues their utterance without a revision. It should be noted that overall, the total number of different words used by both groups was higher each year, which indicates increasing lexical development in Spanish. As expected, children in the DLD group produced fewer words than children in the TLD group.

Despite infrequent use, we analyzed phonological revision mazes. While the HS-DLD showed a significant increase from preschool to first grade, the HS-TLD increased from first to third grade. However, the interpretation of phonological and lexical mazes with code-switching is limited due to the low percentage of these types. Descriptive language sample data revealed that, as a group, children increased their total number of words, number of different words, and mean length of utterance over time, which is consistent with extant language development research even in subtractive language environments (Paradis, 2010; Thordardottir, 2015; Paradis et al., 2021). These results contrast with the idea that exposure to English-only education leads to language loss (Montrul, 2016) and instead show that children continued to develop their language despite the English-only education context (Martinez-Nieto and Restrepo, 2021). Although a few children did undergo language loss, the majority of the children evidenced language growth, especially those with TLD. These results indicate that the language use at home and outside their school contributes to their development, albeit not necessarily at the academic levels expected in monolingual or dual language instruction contexts. Regardless, there may still be transfer of academic skills from the second language to the heritage language.

## Study limitations and future directions

There were several limitations within the current study. There was a relatively small sample size of 22 participants, which limited the power needed to perform more traditional analyses such as ANOVA. Further, not every student had complete data across the three-time points, limiting some of the power. There was also variability in whether full Spanish transcripts were available as students gained English proficiency over time, given that some students refused to provide Spanish samples or produced samples with fewer than 10 Spanish sentences.

The participants in this study were part of a larger sample of HS children (258 total) within an eight-year longitudinal study. Future analyses with these data will include increased sample sizes and compare of maze production in English across all time points (from Preschool to 6th grade). Observations of whether English maze use follows similar or different developmental patterns as children’s change language proficiency changes over time would be of interested to researchers and clinicians.

## Data availability statement

The raw data supporting the conclusions of this article will be made available by the authors, without undue reservation.

## Ethics statement

The studies involving human participants were reviewed and approved by the IRB Arizona State University. Written informed consent to participate in this study was provided by the participants’ legal guardian/next of kin.

## Author contributions

All authors listed have made a substantial, direct, and intellectual contribution to the work, and approved it for publication.
